# E2F8 regulates the proliferation and invasion through epithelial-mesenchymal transition in cervical cancer

**DOI:** 10.7150/ijbs.37686

**Published:** 2020-01-01

**Authors:** Lee Kyung Kim, Sun-Ae Park, Kyung Jin Eoh, Tae-Hwe Heo, Young Tae Kim, Hee Jung Kim

**Affiliations:** 1 Institute of Women's Life Medical Science, Division of Gynecologic Oncology, Department of Obstetrics and Gynecology, Yonsei University College of Medicine, Seoul, 03722, South Korea; 2 Laboratory of Pharmacoimmunology, Integrated Research Institute of Pharmaceutical Sciences, College of Pharmacy, The Catholic University of Korea, Bucheon, 14662, South Korea

**Keywords:** E2F8, invasion, migration, cervical cancer, epithelial-mesenchymal transition

## Abstract

The transcription factor E2F is an important modulator of the cell cycle, and the unrestricted activation of E2F-dependent transcription is considered to be an important driver of tumor formation and progression. E2F8 is known to play an important role in embryonic development and cell cycle control by inhibiting E2F1. However, it is not yet known whether E2F8 is involved in the progression of cervical cancer. In this study, the functional consequences of E2F8 knockdown *in vitro* and *in vivo* were explored. To demonstrate the function of E2F8 in cell proliferation, migration and invasion, we knocked down E2F8 in cervical cancer cell lines; *in vitro* and *in vivo* experiments using this knockdown showed that E2F8 potently induced the expression of epithelial-mesenchymal transition (EMT) markers. Finally, clinical data confirmed that E2F8 was a significant predictive factor for progression-free survival, and that patients with cervical cancer who exhibited high expression of E2F8 showed high FIGO stages and frequent recurrence rates compared to patients with low E2F8 expression. In conclusion, our study suggests that E2F8 is highly correlated with the progression-free survival of cervical cancer patients.

## Introduction

As one of the most common malignant gynecological tumors, cervical cancer is responsible for 10 to 15% of all female cancer related deaths worldwide and is the second leading cause of death from female cancers, exceeded only by breast cancer 1, 2. Moreover, cervical cancer is associated with a high risk of morbidity and mortality due to metastasis and recurrence 3, 4. Therefore, biomarkers of early prognosis, metastasis and novel treatment targets are urgently needed to improve the survival of cervical cancer patients.

Recent studies have shown that the E2F family of transcription factors has many regulatory functions involved in cell proliferation, differentiation, DNA repair, cell cycle and apoptosis 5, 6. So far, the E2F family has eight members, named E2F1 to E2F8. Depending on structure and function, the E2F family can be divided into two groups (standard E2F1-6 and unstructured E2F7-8) and four subgroups (E2F1-3a, E2F3b-5, E2F6 and E2F7-8). There is also evidence that E2F7/8 is involved in the transcriptional suppression of the cell cycle gene for seven or eight years of DNA damage 7, 8. Although the exact role of E2F7 / 8 in DNA damage is not clear, transcription in cell cycle processes that enable optimal DNA recovery is one possibility because high levels of E2F7 / 8 were shown to inhibit proliferation within the laboratory 9. Particularly, recent literature reported that expression of E2F8 was markedly enhanced in multiple carcinomas, which implies that it is involved in oncogenesis and cancer progression 10. However, the biological role and clinical significance of E2F8 in the progression of cervical cancer has not yet been identified in relation to the epithelial-mesenchymal transition (EMT).

In this study, we investigated the functional role of E2F8 in the progression of cervical cancer. Our results show that E2F8 strengthened the growth, migration, and invasion of cervical cancer cells through an EMT signaling pathway. Our study suggests that E2F8 is a promising prognostic factor and target for cervical cancer treatments.

## Materials and Methods

### Patients and tissue samples

In total, 80 women who underwent surgery between 2012 and 2018 at Yonsei Severance Hospital, Yonsei University, seoul, korea, were included in this study. This study was conducted according to the principles of the Helsinki Declaration and ethical guidelines of the Yonsei Severance Hospital Ethics Committee. A sample of newly diagnosed stage IA to IVB cervical cancer (international gynecology and gynecology) was evaluated without prior diagnosis. Additionally, normal cervical tissues from 20 patients undergoing simple hysterectomies because of uterine leiomyomata were obtained as controls. The study was approved by the Yonsei Severance Hospital Ethics Committee and approved by all patients. All samples were immediately frozen in liquid nitrogen until RNA extraction and stored at -80°C.

### Cell lines

Human epithelioid cervical adenocarcinoma (HeLa) cells were obtained from the Korean Cell Line Bank (Seoul, Korea). The epidermoid cervical cancer carcinoma line, established from transfer in small intestine (Caski cells) and ME180 cells, was obtained from the American Type Culture Collection (ATCC, Rockville, MD, USA). Human keratinocyte HaCaT cells were obtained from the Creative Bioarray (Shirley, NY, USA).

HeLa cells were cultivated in Dulbecco's modified Eagle's medium (WelGENE Inc, Korea) and Caski, ME180 and HaCaT cells were cultivated in the RPMI-1640 media (WelGENE Inc., Korea). The culture medium contained 10% (vol/vol) fetal bovine serum and penicillin/streptomycin. The cell lines were maintained at 37°C in humidified air with 5% CO_2_ and 95% air. The passage number of cells was <20 in all experiments.

### Quantitative real-time PCR (qRT-PCR)

RNA was extracted from cancer tissues or cultured cells using TRIzol reagent (Bioline, London, UK). In accordance with the manufacturer's instructions, the total RNA was reversed to cDNA using the reverser reagent kit (Bioline). Real-time PCR analyses were conducted employing a SYBR Green Real-Time PCR Kit (Bioline). Settings for the amplification of E2F8 were as follows: initial denaturation at 95℃ for 3 minutes, 40 cycles with denaturation at 95℃ for 15 seconds, annealing at 60℃ for 60 seconds, and elongation at 72℃ for 60 seconds, then final elongation at 72℃ for 5 minutes. qRT-PCR was accomplished using an ABI StepOnePlus Real-Time PCR System (Applied Biosystems, Foster City, CA, USA). The results were normalized with the expression of U6. The relative change in the expression of mRNA was calculated by the 2-ΔΔCT method. All qRT-PCR experiments were replicated at least three times.

### Small interfering RNA (siRNA) transfection

E2F8 siRNA (siE2F8) and negative control siRNA (siNC) were obtained from Genolution (Genolution Pharmaceuticals Inc., Seoul, Korea). Seeded cells (5 x 10^4^ cells/well) into 6-well plates, using the G-fectin Kit (Genolution Pharmaceuticals) in accordance with the manufacturer's instruction for transfection with 30 nM siRNA in phosphatebuffer saline (PBS). The siRNA-transfected cells were used in *in vitro* assays 48 hours after transfection. The experiments were repeated at least three times.

### E2F8 gene silencing by shRNA

Target shRNA sequences against human E2F8 were designed based on the sequence available from the Gene Bank. The sequence of the E2F8 shRNA was TRCN0000017428: 5'-GCCGCAAAGACAAGTCTTTAA-3'. Oligonucleotides were formed by annealing, then the constructs were cloned into the pLKO.1 vector. HEK293T cells were transfected with shRNA-coded lentiviral vector DNA and packaging vectors (pCMV-VSVG, psPAX2, and pMC2.G) using Lipofectamine 2000 (Life Technologies, Carlsbad, CA, USA). The viral medium was collected at both 48 and 72 hours post-transfection. Cells were infected in the viral medium along with 8 ㎍/ml polybrene, followed by selection with puromycin.

### Cell proliferation assay

The Cell Counting Kit-8 (CCK-8, Dojindo, Japan) assay was used to assess cell proliferation. Cells (5 x 10^4^ cells/well) were seeded into 6-well flat-bottomed plates in 2 mL of complete medium. After incubation of cells overnight to allow for cell attachment and recovery, the cells were transfected with siNC or siE2F8 for 24, 48, 72 or 96 hours. An aliquot of 200 μL of the CCK-8 solution was added to each well and incubated for 1 hour at 37°C. The optical density (OD) was measured at 450 nm using an auto-microplate reader to calculate the number of viable cells in each well. The assay was performed in triplicate.

### Colony formation assay

ME180 cells were transfected with shE2F8 or scrambled (5 x 10^4^) suspensions and then incubated in an upper layer of 1% agar noble (A5431 Sigma-Aldrich, St. Louis, MO, USA) in 2x RPMI with 10% fetal bovine serum. The suspension was overlaid on 0.6% basal agar with 10% fetal bovine serum in a 6-well plate and placed at room temperature until the agarose solidified. The plate was transferred to a 5% CO_2_ incubator, incubated at 37°C for 3 weeks, and stained with crystal violet. Colonies with a diameter greater than 0.5 mm were counted using the ImageJ software (NIH Bethesda, MD, USA).

### Wound healing migration assay

Cell migration was evaluated with a wound healing assay. About 5 x 10^5^ cells were seeded into 6-well plates with serum-containing medium and allowed to grow to 90% confluency in complete medium. The serum-containing medium was eliminated and the cells were serum starved for 24 h. When the cell density reached 100%, artificial homogenous wounds were created by scratching a single layer using a sterile 200 μL pipette tip. After scratching, the cells were washed with phosphate buffer saline (PBS). Using the microscope, cell migration into the wound were captured for 0, 24, and 48 h. The width of the scratch was measured using the NIH ImageJ software and calculated as the percentage of the closed scratch area (width at 0 h / width 48 h). The results were standardized to control cells. The migrated cells were counted in 10 fields under an 20x objective lens. Original magnification as 200x. The experiments were performed in triplicate.

### Transwell migration assay

The migration assay was designed using 6.5 mm diameter Transwell plates (Corning Costar, Cambridge, MA, USA) with 8 μm pore filters. Cells (5 x 10^5^) were seeded in the upper chamber in serum-free medium and complete medium was added to the lower chamber. Cells were incubated for 48 hours in the migration chamber set to 37°C and 5% CO_2_. Non-invasive cells were removed from the upper chamber with a cotton swab. The cells that intruded into the lower surface of the filter were dyed (Diff Quik, Sysmes, Kobe, Japan) and counted with a hemocytometer. This assay was repeated at least three times.

### Matrigel invasion assay

The Matrigel invasion assay was performed using BD Biocoat Matrigel Invasion Chambers (pore size: 8 mm, 24-well; BD Biosciences, Bedford, MA, USA) according to the manufacturer's protocol. Cells (5 x 10^5^) were seeded in the upper chamber in serum-free medium and complete medium was added to the lower chamber. Cells were incubated for 48 h in the Matrigel invasion chamber set to 37°C and 5% CO_2_. Non-invasive cells were removed from the upper chamber with a cotton swab. The cells that entered the lower surface of the filter were stained (Diff Quik, Sysmes, Kobe, Japan) and counted with a hemocytometer. This assay was repeated at least three times.

### Western blot analysis

RIPA buffer (Thermo Fisher Scientific Inc. Waltham, MA, USA) was used to extract proteins. We measured the protein concentrations using a Pierce BCA Protein assay kit (Thermo Fisher Scientific). The protein was boiled with a 5x sample buffer to dissolve it further in a 10% SDS-polyacrylamide gel, followed by electrophoretic transfer to a polyvinylidene difluoride membrane (Milipore, Billica, MA, USA). After blocking with 5% fat-free dried milk in 1x Tris-buffered saline containing 0.1% tween 20 (pH 7.6) at room temperature for 1 hour, the membrane was continuously stirred and incubated with primary antibody at 4℃ overnight.

### Xenografts in mice

BALB/c mice (n = 30, 5-6 weeks of age, Orient Bio, Seongnam, Korea) were kept in aseptic conditions under constant temperature and humidity (Yonsei Medical University protocol). Each mouse received a subcutaneous dorsal scapula injection of a 200 μL suspension of HeLa cells pretreated with siE2F8 and siNC for 48 h (1.0 x 10^6^ cells/flank, xenograft n=18). ME180 cells pretreated with shE2F8 or shscrambled control were transplanted (2.0 x 10^6^ cells/flank, xenograft n=12) into the subcutaneous dorsal scapula injection.

Calipers were used to measure the tumor sizes twice a week. The tumor volume was calculated using a simplified equation to estimate a rotational ellipsoid (length x width^2^ x 0.5). Each tumor was harvested 40 days post-treatment.

### Magnetic resonance (MR) imaging in mice

MR images were obtained using Brucker Biospec 94/24 USR (9.4T) small animal scanner (35 mm diameter cage coil, Brut BioSpin MRI, Ettlingen, Germany). A custom-built cradle was used to immobilize each mouse during the MR imaging process. At the beginning of each imaging session, the T2-weighted image was obtained using the quick acquisition setting. These images were used to confirm that the animal was in the correct position inside the magnetic bore. A 1.5% isoflurane and O_2_/N_2_O (1:1) mixture at a 0.7 L/min flow rate was used as anesthesia during the MR imaging. Breathing was monitored using an air pillow. The body temperature of the mice was maintained within acceptable limits using circulating warm water.

### Hematoxylin and eosin (H&E) staining

Mice were euthanized after the MRI. Tumor tissue was collected, fixed with 4% paraformaldehyde for 24 h, washed in PBS, and then embedded in paraffin. Two-micrometer sections were stained with hematoxylin and eosin following standard procedures.

### Statistical analyses

IBM SPSS version 24 for Windows (SPSS Inc., Chicago, IL, USA) was used for statistical analyses. We used Pearson's χ^2^ tests, Student's *t*-tests, and Fisher's exact tests to evaluate the associations between E2F8 expression and clinicopathological characteristics. In order to evaluate the performance of the model with respect to their discriminating capability, the chi-square value of the log rank test was used in the receiver operating characteristic (ROC) analysis. The median value (1.2962) was set to the cutoff value. These groups were classified as high and low E2F8 expression groups, respectively, at the above and below values of the cut-off value of 1.2962. The entire survival time was analyzed using the Kaplan-Meier method. The log rank test was used to estimate the differences between groups. The stepwise Cox proportional hazards model was used for multivariate survival analysis of the parameters which were significant in the univariate analysis. The statistical tests were two-sided and p-values of less than 0.05 were considered significant.

## Results

### E2F8 expression was elevated in cervical cancer and correlated with poor prognosis

**Figure 1:**
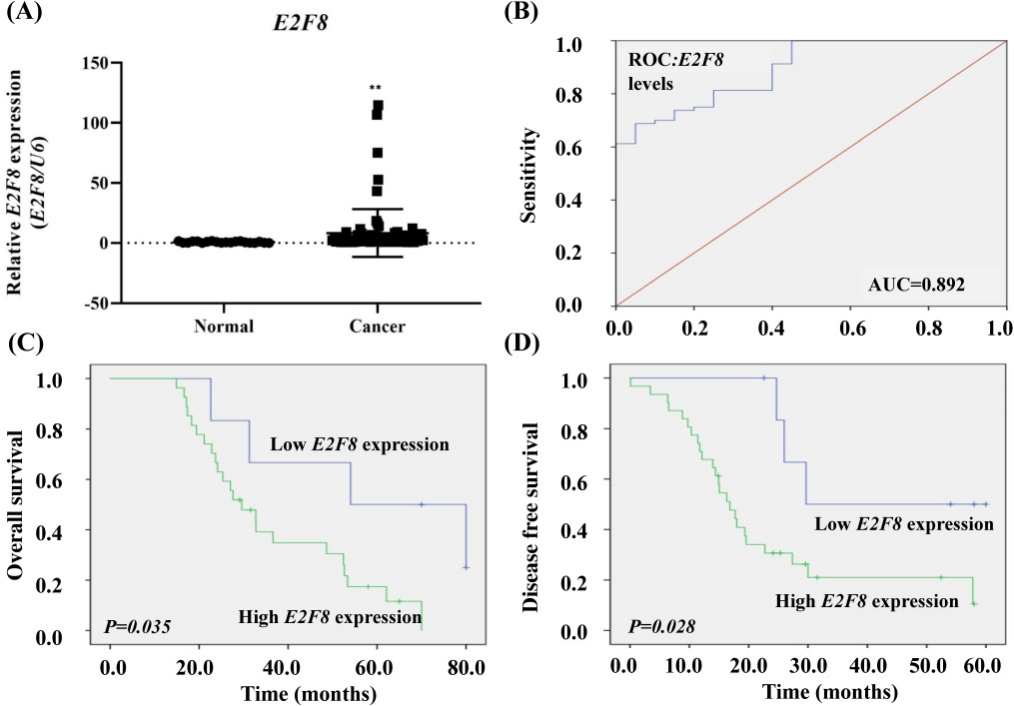
** The clinical significance of *E2F8* expression in cervical cancer tissue. (A)** The *E2F8* expression was significantly higher in cervical cancer tissues (n=80) than in non-cancerous tissues (n=20). *E2F8* expression was determined using qRT-PCR and is expressed relative to the control value. Data are expressed as mean ± SD. ^* *^ *p*<0.01 vs. non-tumor control. **(B)** ROC curve for prognostic predictions for patients by *E2F8* levels. The AUC is shown in the plots. ROC, receiver operating characteristic; AUC, area under the curve. **(C)** Kaplan-Meier curves for the overall survival and **(D)** disease-free survival of cervical cancer patients with different expression levels of *E2F8.*

To determine whether E2F8 expression in tissues was associated with the clinicopathologic characteristics of cervical cancer, we evaluated the expression of E2F8 in cervical cancer tissue (n =80) and corresponding normal tissue (n =20). The E2F8 expression in cervical cancer tissues was more than 11.07 times that of non-cancerous tissues (*p*= 0.001; Figure 1A).

The risk model for E2F8 data showed a predicted AUC of 0.892 (*p*= 0.00000007; Figure 1B.). We compared the characteristics of patients with high E2F8 expression (n=62) to those with low E2F8 expression (n=18; Table S1). Kaplan-Meier survival analysis demonstrated that cervical cancer patients with low E2F8 levels exhibited longer overall survival and disease-free survival than cervical cancer patients with high E2F8 levels (*p*=0.035 and 0.028, respectively Figure 1C and D).

Moreover, according to univariate and multivariate analyses using the Cox proportional hazards model, E2F8 expression was a significant predictive factor for stage and recurrence (stage, univariate hazard ratio (HR)= 1.844 (1.276-2.663), *p*=0.001, multivariate HR=1.816 (1.258- 2.622), *p*=0.001; recurrence, univariate HR=7.545 (3.535-16.103), *p*=0.000000017, multivariate HR= 7.823 (3.605-16.973), *p*=0.0000001; Table 1). Both univariate and multivariate proportional hazard analyzes showed that stage and recurrence were independent prognostic factors for overall survival.

### E2F8 expression was elevated in cervical cancer cell lines and correlated with cell proliferation

**Figure 2:**
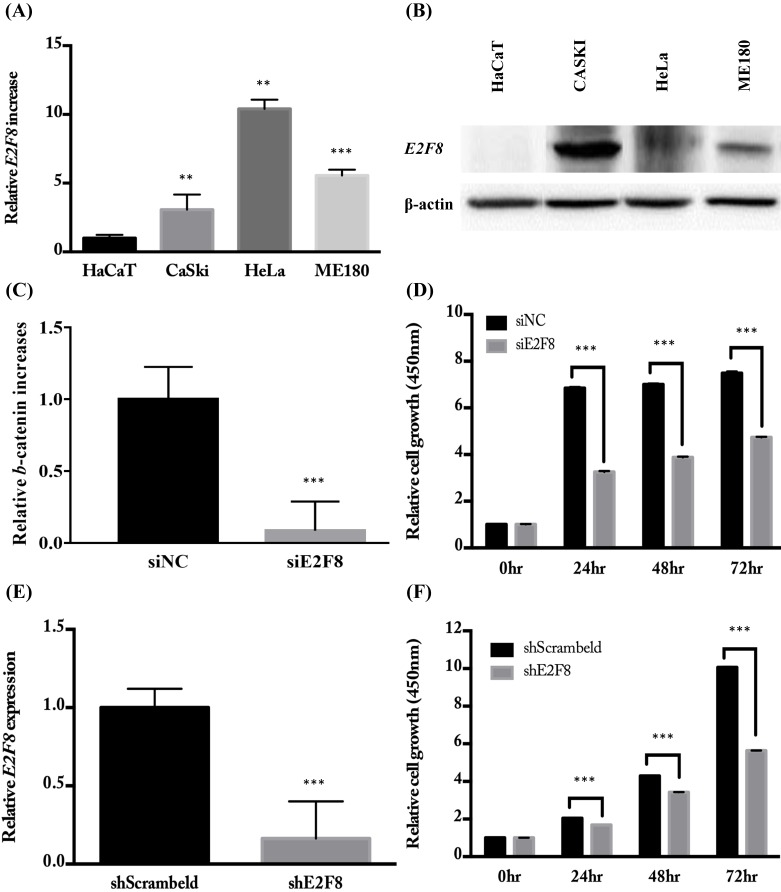
** E2F8 expression in cervical cancer cell lines and correlation with suppressed cell proliferation. (A, B)** The expression levels of E2F8 RNA and protein were significantly higher in some cervical cancer cell lines (CaSki, HeLa, and ME180) compared to that in normal control cells. **(C)** Using siE2F8, E2F8 knockdown was performed in E2F8-elevated HeLa cell lines. **(D)** The proliferation of HeLa cells transfected with siE2F8 and negative control siNC was determined using the CCK-8 assay. **(E)** Using shE2F8, E2F8 knockdown was performed in E2F8-elevated ME180 cell lines. **(F)** The proliferation of ME180 cells transfected with shE2F8 and negative scrambled control was determined using the CCK-8 assay. Each assay was performed in triplicate. Data are means ± standard deviation. **P<0.01, ***P<0.001 vs siNC and scrambled control.

To investigate the E2F8 expression in cervical cancer cells, several cell lines were examined for mRNA and protein levels of E2F8 (Figure 2A and B). As shown in Figure 2, CaSki, HeLa, and ME180 cells expressed higher levels of E2F8 than the control (HaCaT) cells. Next, we examined the effects of E2F8 in cervical cancer cells. By transfecting siE2F8 and shE2F8, E2F8 was knocked down in HeLa and ME180 cells. The siE2F8 and shE2F8-transfected HeLa and ME180 cells also showed significant inhibition of cell proliferation (Figure 2C and E). E2F8 inhibited cell proliferation by 50% and 60% at 72 h post-transfection in HeLa and ME180 cell lines, respectively, relative to control cells (Figure 2D and F). Thereafter, colony formation assays confirmed that E2F8 inhibited cell colony formation and by 90% in ME180 cell lines relative to control cells (Figure 2G and F).

### E2F8 knockdown inhibited the migration and invasion of cervical cancer cells

**Figure 3:**
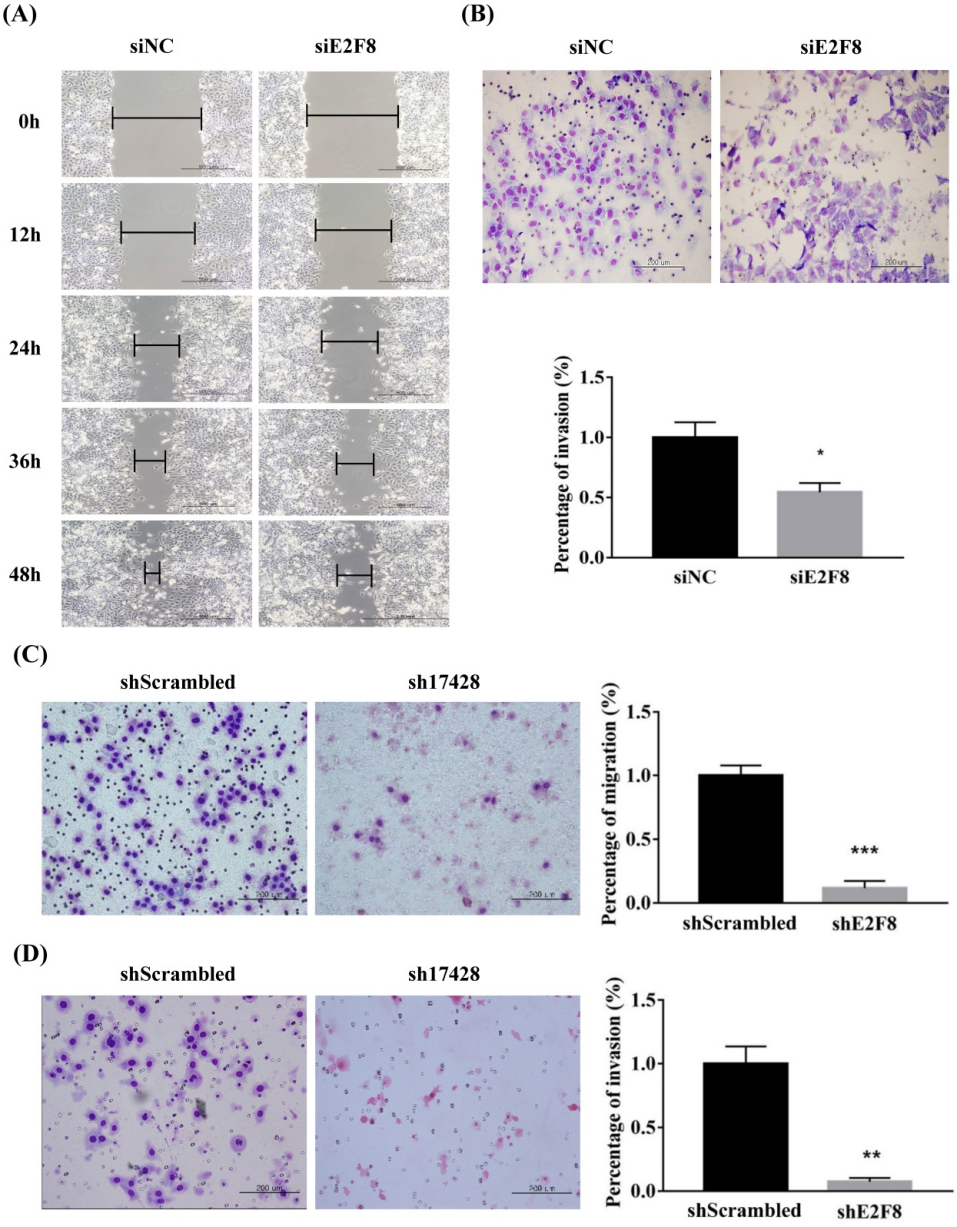
** E2F8 promotes cell migration and invasion. (A, C)** Wound healing assay observed under the optical microscope was used to determine cell migration using si and shE2F8. E2F8 knockdown was performed in E2F8-high HeLa and ME180 cell lines. **(B, D)** Cell invasion was observed under the optical microscope. Matrigel invasion assays were used to determine invasion after 48 h in HeLa and ME180 cells. Each assay was performed in triplicate. Data represent means ± standard deviation. **P<0.01, ***P<0.001 vs. siNC and scrambled control.

We investigated whether E2F8 affects the invasion and migration of cervical cancer cells. A Matrigel invasion assay was used to assess invasion after 48 h. E2F8 knockdown in cervical cancer cell lines (HeLa and ME180) showed significant decrease in wound-healing. Therefore, the same cell lines were used to assess migration in si- and sh-E2F8 transfected cervical cancer cells (Figure 3A and C). In addition, the invasiveness of these cells was compared to that of control cells. The E2F8 knockdown cells exhibited reduced invasiveness (Figure 3B and D). While uncontrolled cell proliferation is a common biological feature of all tumors, the key pathophysiological feature of malignant tumors in pathological conditions is the ability to penetrate the natural tissue barrier. Our findings suggest that E2F8 expression correlated with cancer invasion and metastasis**.**

### E2F8 knockdown reversed EMT signaling pathway-related genes in cervical cancer cells

Because EMT is crucial in cell migration and invasion, the identification of factors associated with EMT can have a clinical impact 11, 12. Thus, we examined whether E2F8 was associated with EMT. Towards this, EMT-related markers were evaluated using real-time RT-PCR (Figure S1A and 4A) and Western blotting (Figure S1B and 4B) after the si- and sh-RNA-mediated knockdown of E2F8 in HeLa and ME180 cells. Knockdown of E2F8 increased E-cadherin expression and decreased the expression of N-cadherin, b-catenin, vimentin, Wnt5, and Twist. These results indicate that the dysregulation of EMT-related genes may explain the involvement of E2F8 in cervical cancer cell migration and invasion.

### E2F8 knockdown blocked tumor growth in a xenograft nude mouse model

**Figure 5:**
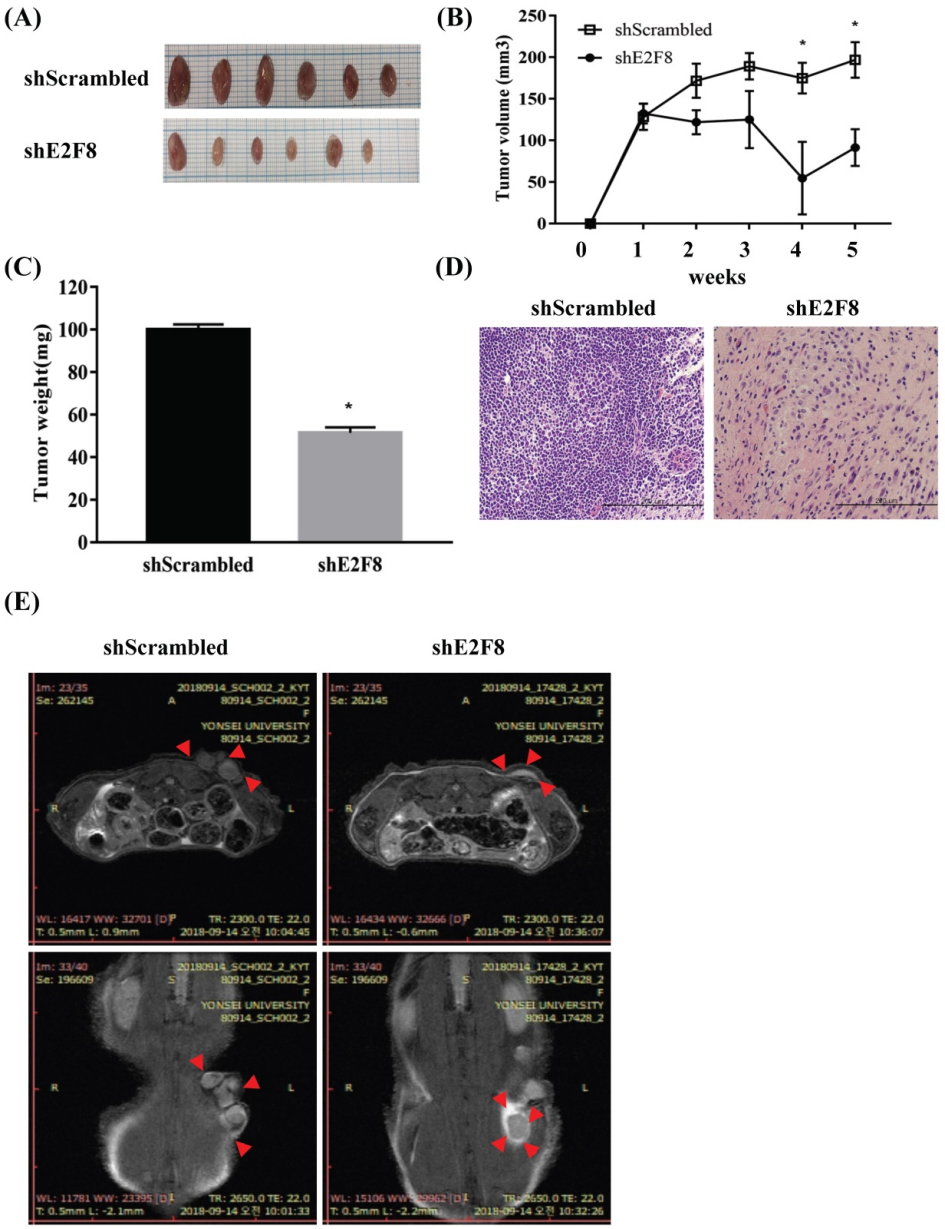
** E2F8 knockdown decreases tumor size in a xenograft nude mouse model. (A)** ME180 cells transfected with shE2F8 or scrambled control were injected subcutaneously into the right dorsal scapula area of nude mice. Gross images of tumor masses from representative mice from each group. **(B)** Tumor sizes in the experimental groups. **(C)** Tumor weights were compared after tumor harvest. **(D)** Hematoxylin and eosin (H&E) staining of shE2F8-transfected ME180 cells (x200). (E) MRI image. *p<0.05 vs. scrambled control.

To explore whether E2F8 affected tumor growth *in vivo*, we implanted HeLa and ME180 cells, in which E2F8 was knocked down, as xenografts into nude mice (Figure S2A and Figure 5A). Tumor volume (Figure S2B and Figure 5B) and weights (Figure S2C and Figure 5C) were measured. The mean tumor volumes and weights of mice, which were implanted with siE2F8 transfected HeLa cells and shE2F8 transfected ME180 cells were significantly smaller than those of mice that had been impalnted with si- and sh-scrambled control cells (*p* < 0.05. Histological examination revealed that more cells with small nucleoli and irregular nuclear membranes were present in the E2F8 knockdown xenografts than that in the control xenografts (Figure S2D and Figure 5D). We further evaluated tumor size and activity using magnetic resonance imaging (MRI) (Figure S2E and Figure 5E). These findings suggested that E2F8 promoted tumor growth *in vivo*, and further supported our hypothesis that E2F8 is involved in the malignant transformation of cervical cancer cells.

## Discussion

The deregulation of E2F- dependent transcription occurs frequently in many cancers and is considered an important factor in the uncontrolled proliferation of cancer cells. However, the function and contribution of E2F family members in various types of cancers is complex and poorly understood 13. Most studies have indicated that E2F deregulation in tumors is a result of overexpression of proliferation‐promoting transcription factors, rather than deregulation of the mainly inhibitory transcription factors. In non-small cell lung cancer and esophageal squamous cell carcinoma, tumor progression and poor outcomes correlate with overexpression of the proliferation‐promoting transcription factor E2F1 14, 15. In contrast, tumor growth is enhanced by the downregulation of inhibitory E2F transcription factors such as E2F4 and E2F7, and such an imbalance may cause a switch from cell quiescence to growth promotion 16. E2F protein is a proven modulatory factor in malignant progression in various cancers. The newly identified E2F8 has been reported to be an important proliferation factor in some human cancers 17. A deeper understanding of the molecular mechanisms that determine the progression and metastasis of cervical cancer is essential for the development of more effective therapies and for the identification of new diagnostic indicators of cervical cancer.

Recent studies have shown that some abnormal molecular changes can play a central role in the development of cervical cancer 18. However, the clinical significance and biological function of E2F8 in gynecological cancer, especially cervical cancer, remains unknown. Accordingly, in this study, the molecular function and clinical significance of E2F8 expression was investigated in cervical cancer cell lines. We found that E2F8 expression was higher in cervical cancer tissues than that in non-cancerous tissues. Moreover, E2F8 knockdown altered the cell growth, migration, and invasion of cervical cancer cells. The metastatic effects of E2F8 appeared to be mediated, at least in part, by regulation of the genes involved in cell migration, invasion, and EMT.

There is some evidence that E2F8 might also be associated with human cancers. An increase in the E2F8 gene copy number was detected in melanoma 19 and increased expression was reported in ovarian cancer and hepatocellular carcinoma 20. E2F8 knockdown caused an almost complete block of cell proliferation and a substantial induction of apoptosis, but did not inhibit normal bronchial epithelial cell growth. These results indicate that while E2F8 does not serve a role in normal cells, it plays an integral role as a tumor promoter in cancer cells.

To better understand the direct role of E2F8 in carcinogenesis, we targeted EMT signaling by knockdown of E2F8. The EMT involves alterations in cell phenotypes and several transcription factors have been implicated in the regulation of EMT-related gene expression. Although several studies have focused on transcriptional regulators in pathological EMT, few studies have evaluated the roles of transcription factors in cervical cancer 21. The present study explored the molecular function of E2F8 expression in cervical cancer cell lines. Our findings showed that downregulated E2F8 expression was correlated with decreased cell growth, migration, and invasion of cervical cancer cells. This effect of E2F8 on tumor progression may be mediated by genes involved in cell migration, invasion, and the EMT signaling pathway, as well as genes that encode E-cadherin, N-cadherin, β-catenin, vimentin, and Snail. Our findings suggested that E2F8 could potentially represent a novel biomarker and therapeutic target for cervical cancer. The loss of E-cadherin is thought to be an important event in the EMT, but N-cadherin reduces the intercellular connection between two adjacent endothelial cells, causing the cancer cells to slip 22. In addition, β-catenin is more slowly mobile and weakens the related mesenchymal phenotype. The improvement in the expression of transcription factors, such as in snail and twist, is related to the loss of adhesion between cells 23. Vimentin is the main component of the cytoskeleton of mesenchymal cells and its upregulation is induced by the EMT 24, 25.

We hypothesized that E2F8 may act as an important regulator for several signaling mechanisms related to the EMT. Our results suggest that E2F8 can contribute to the growth, invasion, and recurrence of cervical cancer through induction of the EMT. The recurrence rate of advanced cervical cancer after radical surgery is 15 to 30% and the prognosis of patients with recurrence is poor 26. In order to improve the prognosis of cervical cancer patients, a firm prediction of relapse and progression is needed. The EMT signaling pathway contributes to tumor progression characteristics, including invasion, metastasis, and angiogenesis 27. Furthermore, clinical outcome data from 80 patients with epithelial cervix cancer support the findings of our *in vitro* experiments.

In summary, our research suggests that E2F8 is correlated with the progression of cervical cancer via EMT signaling pathways. These results support E2F8 as a promising prognostic marker and therapeutic target for cervical cancer.

## Supplementary Material

Supplementary figures and tables.

## Figures and Tables

**Figure 4:**
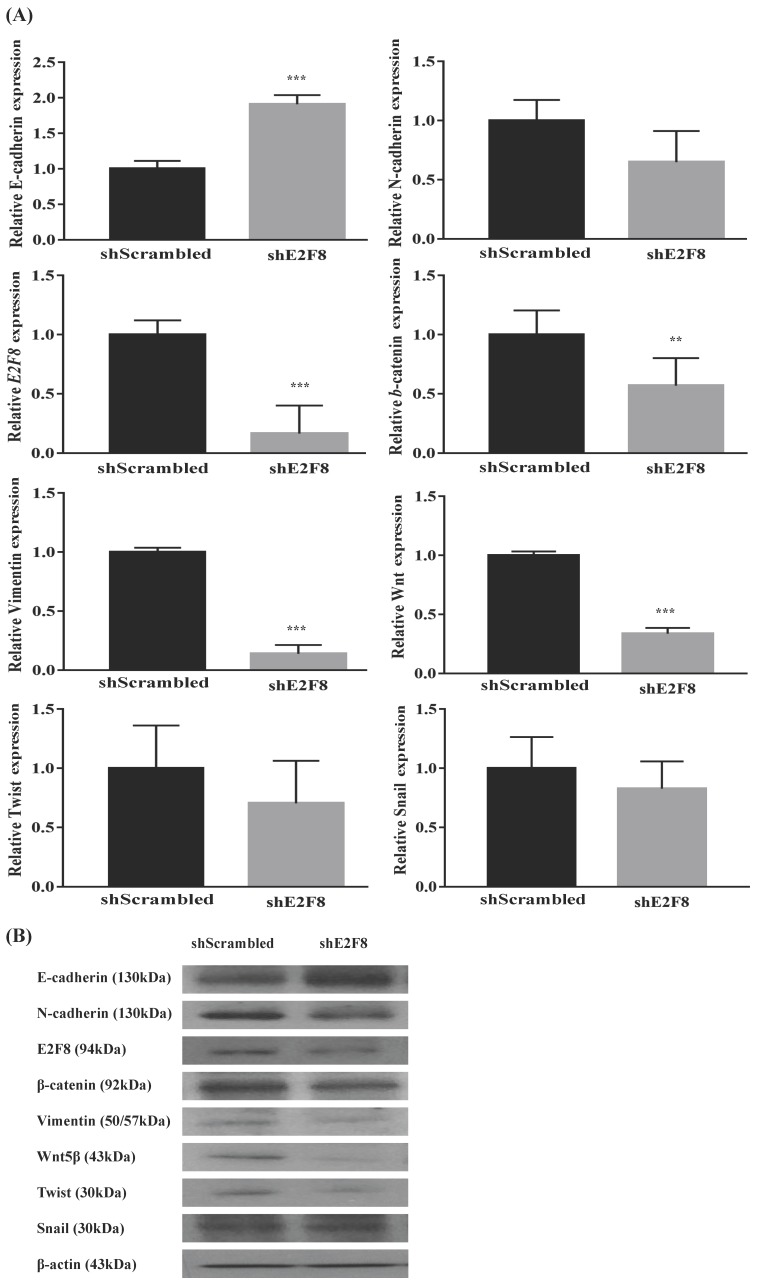
** Effect of E2F8 Knockdown on EMT progression. (A)** ME180 cells were transfected with shE2F8 or scrambled control. Real-time RT-PCR of EMT-related transcription factors after E2F8 knockdown in ME180 cells. Data represent means ± SD. **P<0.01, ***P<0.001 vs. scrambled control. **(B)** Protein lysates were prepared using shE2F8- and scrambled control-transfected ME180 cells.

**Table 1:** Univariate and multivariate analysis of various factors for overall survival

	OS				
	Univariate analysis			Multivariate analysis	
	HR (95% CI)	P		HR (95% CI)	P
**E2F8 expression**	1.108 (0.481-2.556)	0.809			
**Age, years (continuous)**	1.003 (0.974-1.033)	0.837			
**FIGO stage**	1.844 (1.276-2.663)	0.001		1.816 (1.258-2.622)	0.001
**cell type**	1.026 (0.720-1.461)	0.889			
**Lymph node metastasis**	1.939 (0.977-3.847)	0.054			
**Lymphovascular invasion**	1.385 (0.658-2.915)	0.39			
**Recurrence**	7.545 (3.535-16.103)	0.000000017		7.823 (3.605-16.973)	0.000000195
**tumor size**	1.365 (0.849-2.195)	0.199			

OS, overall survival; HR, hazard ratio; FIGO, The International Federation of Gynecology and Obstetrics

## Abbreviations

E2F8: the E2 factor family 8
EMT: epithelial- mesenchymal transition
CCK-8: cell counting kit-8
