# Supplementary material for: E2F8 regulates the proliferation and invasion through epithelial-mesenchymal transition in cervical cancer
Source: Int J Biol Sci. 2020 Jan 1;16(2):320–9. doi: 10.7150/ijbs.37686 (PMC6949145; doi:10.7150/ijbs.37686)
Supplement: Supplementary file 1 — Supplementary figures and tables. [file ijbsv16p0320s1.pdf]

Table S1. Patient characteristics

Factor		N (%)	E2F8 expression	
			Low	High
Age (mean±SD)		80	48.11±9.99	50.37±12.28
Stage				
	I	27	7	20
	II	16	4	12
	III	27	5	22
	IV	10	2	8
Lymphnode metastasis				
	Yes	49	10	39
	No	31	8	23
Lymphatic invasion				
	Yes	19	5	14
	No	61	13	48
Tumor size				
	<2cm	16	3	13
	2_4	10	2	8
	≥4cm	54	13	41
Recur				
	Yes	28	4	24
	No	52	14	38
cell type				
	Squamous cell carcinoma	52	14	38
	Adenocarcinoma	15	3	12
	Mixed	6	0	6
	Other	7	1	6

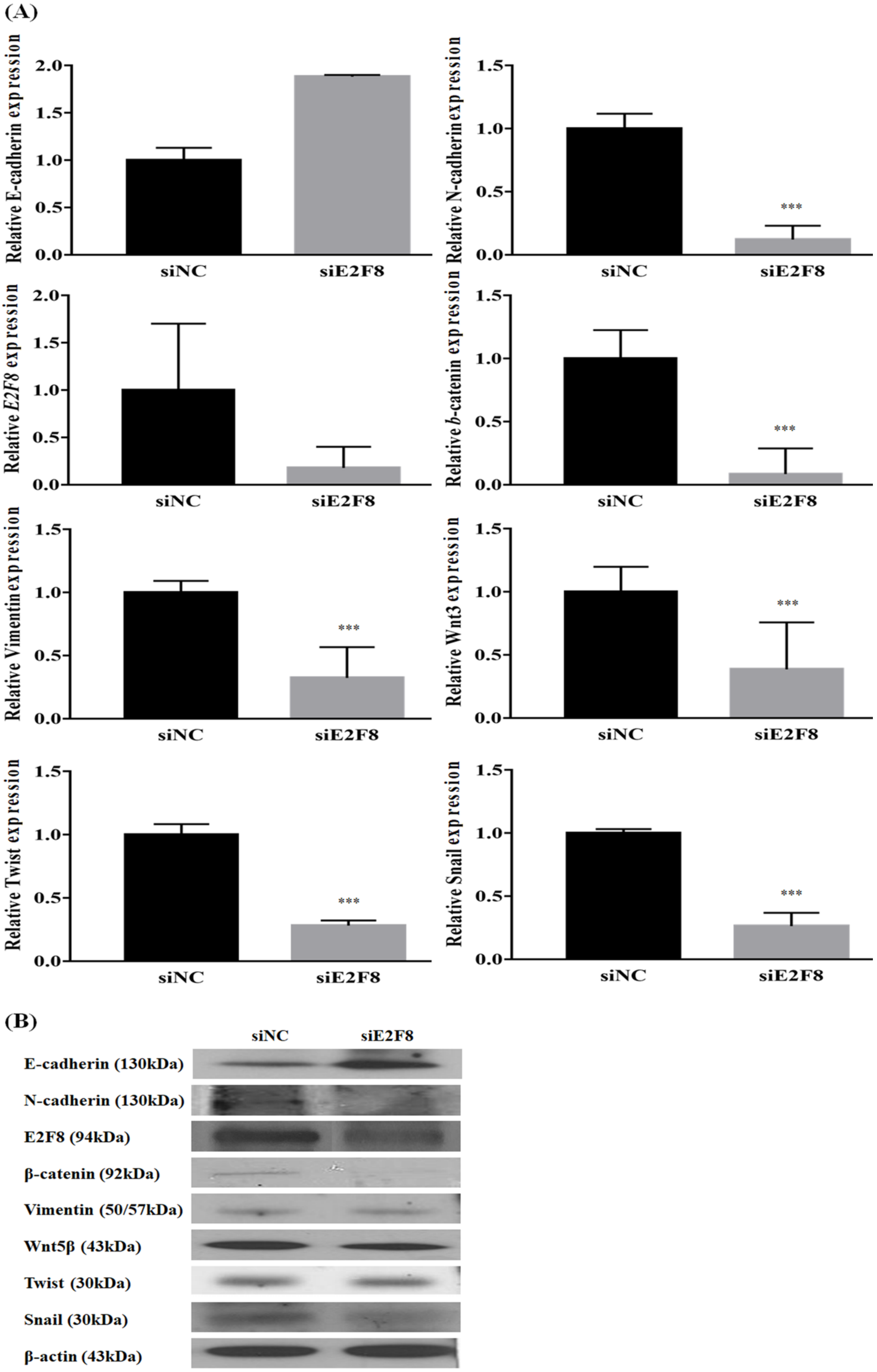

(A)
Relative E-cadherin expression
2.0
1.5
1.0
0.5
0.0
siNC
siE2F8
Relative N-cadherin expression
1.5
1.0
0.5
0.0
***
siNC
siE2F8
Relative E2F8 expression
2.0
1.5
1.0
0.5
0.0
siNC
siE2F8
Relative b-catenin expression
1.5
1.0
0.5
0.0
***
siNC
siE2F8
Relative Vimentin expression
1.5
1.0
0.5
0.0
***
siNC
siE2F8
Relative Wnt3 expression
1.5
1.0
0.5
0.0
***
siNC
siE2F8
Relative Twist expression
1.5
1.0
0.5
0.0
***
siNC
siE2F8
Relative Snail expression
1.5
1.0
0.5
0.0
***
siNC
siE2F8
(B)
siNC
siE2F8
E-cadherin (130kDa)
N-cadherin (130kDa)
E2F8 (94kDa)
β-catenin (92kDa)
Vimentin (50/57kDa)
Wnt5β (43kDa)
Twist (30kDa)
Snail (30kDa)
β-actin (43kDa)

Figure S1. Effect of E2F8 Knockdown on the EMT progression.

(A) HeLa cells were transfected with siE2F8 and siNC for 48h. Real-time RT-PCR of EMT-related transcription factors after E2F8 knockdown in HeLa cells. Data represent means ± SD. **P<0.01, ***P<0.001 vs. siNC. (B) Protein lysates were prepared using siE2F8 and siNC transfected HeLa cells 48h post-transfection

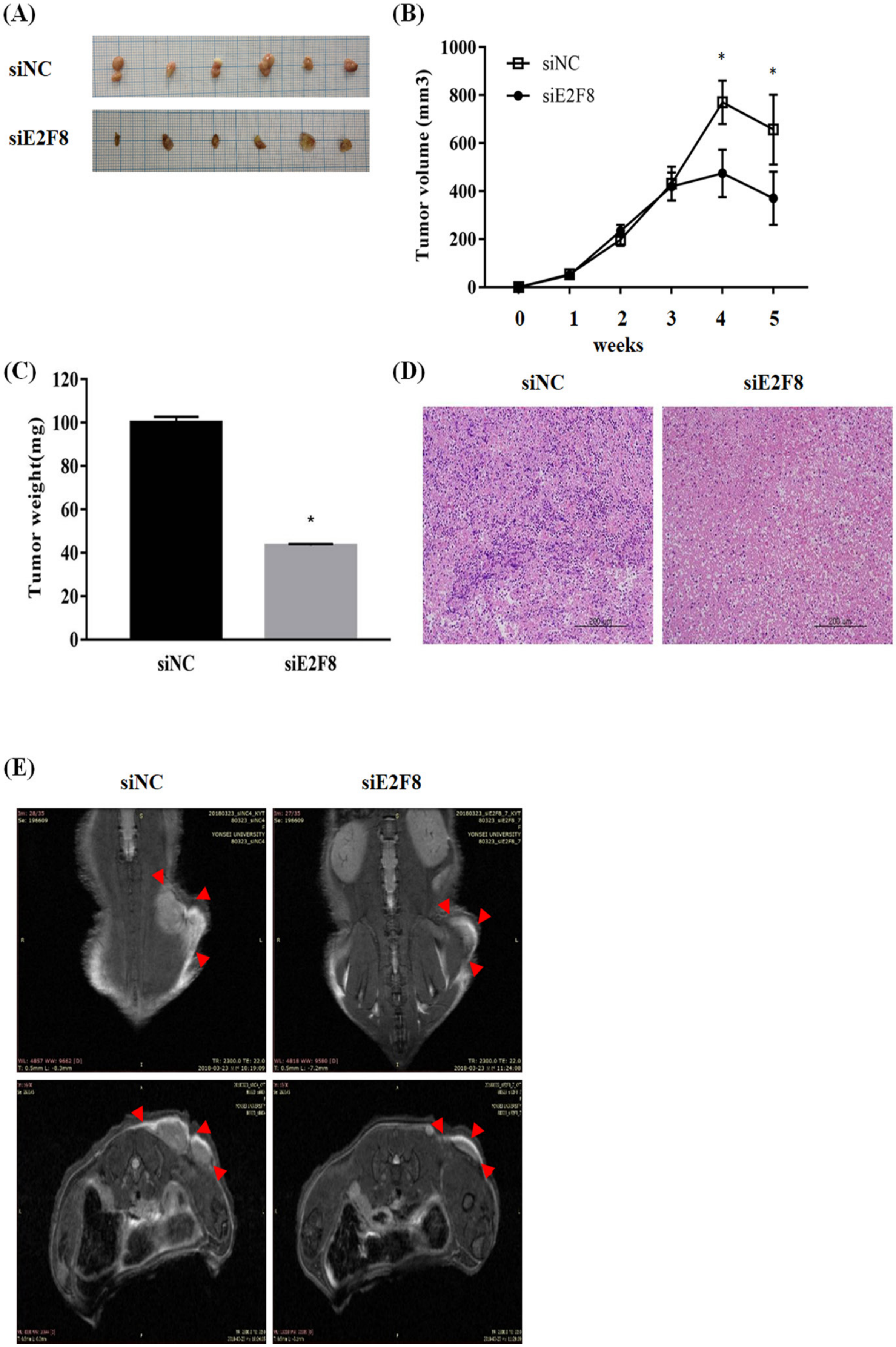

(A)
siNC
siE2F8
(B)
Tumor volume (mm3)
1000
800
600
400
200
0
siNC
siE2F8
*
*
0
1
2
3
4
5
weeks
(C)
Tumor weight(mg)
120
100
80
60
40
20
0
*
siNC
siE2F8
(D)
siNC
siE2F8
(E)
siNC
siE2F8
YONSEI UNIVERSITY

Figure S2. E2F8 knockdown decreases tumor size in a xenograft nude mouse model. (A) HeLa cells transfected with siE2F8 and siNC were injected subcutaneously dorsal scapula into the right tumor of nude mice. Gross images of tumor masses from representative mice from each group. (B) Tumor sizes in the experimental groups. (C) Tumor weights were compared after tumor harvest. (D) Hematoxylin and eosin (H&E) staining of siNC, and siE2F8-transfected HeLa cells (x200). (E) MRI image. *$p<0.05$, ***$p<0.001$ vs. siNC
